# Early-Life Exposure to DDT from Indoor Residual Spraying and Adult Risk of Reproductive Cancers: A Nationwide Study with Long-Term Follow-Up in Taiwan

**DOI:** 10.3390/cancers18111816

**Published:** 2026-06-01

**Authors:** Ya-Chi Chang, Yu-Yin Chang, Wei-Te Wu, Pau-Chung Chen

**Affiliations:** 1 Institute of Environmental and Occupational Health Sciences, College of Public Health, National Taiwan University, Taipei 10055, Taiwan; ycchang@jpinlaw.com.tw; 2 National Institute of Environmental Health Sciences, National Health Research Institutes, Miaoli 35053, Taiwan; yuyin.chang@gmail.com (Y.-Y.C.); wade.wu@nhri.edu.tw (W.-T.W.); 3 Department of Environmental and Occupational Medicine, National Taiwan University Hospital and National Taiwan University College of Medicine, Taipei 10048, Taiwan

**Keywords:** DDT, indoor residual spraying, early-life exposure, reproductive cancers, testicular cancer, cervical cancer, cancer stage, DOHaD

## Abstract

**Background:** Early-life exposure to dichlorodiphenyltrichloroethane (DDT) may increase adult cancer risk, but evidence from Asian populations remains limited. Taiwan’s nationwide indoor residual spraying (IRS) program during the 1950s provides a unique setting to examine long-term reproductive cancer risk associated with early-life DDT exposure. **Methods:** We conducted an ecological study using township-level DDT IRS frequency (0–5 times) as the exposure indicator. Individuals born between 1952 and 1958 were followed from 1979 to 2022 for incident reproductive cancers based on data from the Taiwan Cancer Registry. Poisson regression models were applied to estimate relative risks associated with each additional IRS exposure. **Results:** A total of 109,244 reproductive cancer cases were identified. Each additional DDT spraying round was associated with increased risks of breast, ovarian, corpus uteri, prostate, testicular, and cervical cancers (RRs = 1.01–1.16). Elevated risks were observed for testicular cancer (RR = 1.16, 95% CI: 1.01–1.23) and cervical cancer (RR = 1.01, 95% CI: 1.002–1.02), for which Asian epidemiological evidence remains limited. Higher exposure levels were also associated with differences in stage at diagnosis for breast cancer among women aged ≥55 years and for corpus uteri cancer. **Conclusions:** Early-life DDT exposure was associated with increased risks of several reproductive cancers. These findings support the Developmental Origins of Health and Disease framework and suggest that environmental exposures during critical developmental windows may influence long-term cancer risk. However, the findings should be interpreted cautiously given the ecological study design and potential residual confounding.

## Simple Summary

Exposure to endocrine-disrupting chemicals during early life may have lasting effects on health. In Taiwan, a nationwide indoor DDT spraying program was implemented in the 1950s for malaria control, resulting in widespread exposure among pregnant women and young children. This study investigates whether early-life exposure to DDT is associated with an increased risk of hormone-related cancers in adulthood. Using long-term population-based data, we aim to clarify how exposures during critical developmental periods influence cancer risk later in life. The findings provide important evidence on the long-term health effects of environmental chemicals and offer valuable insights for researchers and policymakers in evaluating the safety and public health trade-offs of pesticide use.

## 1. Introduction

During the mid-20th century, dichlorodiphenyltrichloroethane (DDT) became one of the most important vector-control agents worldwide, particularly in malaria eradication programs, and contributed substantially to saving millions of lives [1]. However, subsequent studies have gradually revealed its potential adverse effects on the environment and human health, leading to restrictions or bans in most countries since the 1970s [1]. Nevertheless, due to its high environmental persistence and lipophilicity, DDT readily accumulates in ecosystems and human adipose tissue, resulting in continued human exposure and potential long-term health effects even decades after its peak use [1,2].

Vector control remains a critical component of public health strategies in many endemic regions, as environmental conditions, climate variability, and land-use patterns continue to influence the transmission of vector-borne diseases [3]. Although pesticides remain necessary in certain settings, concerns persist regarding their environmental persistence and potential human health effects [4]. Moreover, despite being banned or restricted in most countries, DDT continues to be used in some malaria-endemic regions under World Health Organization (WHO) guidelines, making its long-term health impacts an ongoing global public health concern [5].

DDT is classified as an endocrine-disrupting chemical (EDC) with both estrogenic and anti-androgenic properties [6,7]. Mechanistically, DDT mimics endogenous estrogen to promote cell proliferation in hormone-sensitive tissues [6], while its metabolite DDE interferes with androgen receptor signaling, potentially increasing risks for prostate and testicular cancers [7]. Beyond hormonal disruption, DDT exposure can induce oxidative stress, DNA damage, and epigenetic reprogramming, such as abnormal DNA methylation [8,9,10]. Furthermore, it may promote carcinogenesis through chronic inflammatory responses and disruption of intercellular signaling, thereby altering the tumor microenvironment and facilitating cancer progression [8,11,12].

However, epidemiological evidence regarding the association between DDT exposure and cancer risk remains inconsistent. While some studies have reported positive associations between DDT/DDE exposure and the risk of breast and prostate cancers, several meta-analyses have found no significant associations [13,14]. These inconsistencies may be related to the fact that most studies measured serum exposure levels during adulthood and therefore failed to capture critical early-life exposures [10,15]. In addition, substantial variation in exposure levels across studies and differences in susceptibility among populations with different genetic backgrounds may also contribute to the inconsistent findings [8]. Therefore, reliance solely on adult exposure data may underestimate the true carcinogenic potential of DDT, highlighting an important evidence gap in current environmental health research.

In Taiwan, the incidence of endocrine-related cancers has surged over recent decades. According to the Taiwan Cancer Registry Annual Report, breast cancer and prostate cancer ranked first and sixth among the top ten cancers in 2003, with age-standardized incidence rates (ASIRs) of 38.7 and 14.3 per 100,000 population, respectively [16]. By 2023, the ASIRs had increased to 102.7 for breast cancer and 37.2 for prostate cancer, with prostate cancer rising to the third most common cancer. In addition, corpus uteri, thyroid, and ovarian cancers also entered the top ten cancer rankings [17], reflecting a broader shift in the national cancer burden.

The increasing incidence of endocrine-related cancers has raised concerns regarding the association between exposure to environmental chemicals and cancer development [18], particularly during critical developmental periods [18,19]. Compared with exposures occurring during adulthood, even very low-level exposure to endocrine-disrupting chemicals (EDCs) during early life may be sufficient to disrupt hormonal balance and result in long-term and potentially irreversible health effects [18]. Based on this understanding, Taiwan’s nationwide indoor residual spraying (IRS) campaign using DDT during the 1950s provides a unique opportunity to investigate whether individuals born during this period may have an increased risk of developing endocrine-related cancers later in life.

During the 1950s, in response to the World Health Organization’s Global Malaria Eradication Programme [20], Taiwan implemented a nationwide IRS campaign beginning in 1952 and ending in 1957. Supported by U.S. funding and WHO technical assistance, this six-year initiative was not uniform; the frequency of DDT application varied across townships according to malaria severity, resulting in an exposure gradient ranging from 0 to 5 applications [21,22]. This distinctive historical context constitutes a natural experiment, generating spatial variation in exposure intensity and enabling comparisons of long-term health outcomes across populations with different levels of early-life exposure.

In our previous study, we established a retrospective cohort of individuals born between 1952 and 1958 based on this historical setting and linked it with Taiwan’s National Health Insurance Research Database to investigate the risk of overall cancer incidence in adulthood [23]. The results showed that early-life exposure to DDT was associated with an increased risk of several hormone-related cancers, including breast, ovarian, corpus uteri, and prostate cancers [23]. These findings highlight the importance of critical windows of susceptibility in disease development. However, because Taiwan’s National Health Insurance system was not implemented until 1995, the previous study was limited to cancer cases identified in the insurance database between 2000 and 2022, potentially underestimating cancers with earlier onset, such as testicular cancer, and precluding analyses of cancer stage at diagnosis.

To address these limitations, the present study utilized data from the Taiwan Cancer Registry (TCR), which has systematically collected nationwide cancer incidence data since 1979, allowing us to track cancer occurrence among participants from 1979 to 2022. Building upon our previous findings, the present study specifically focuses on reproductive system-related cancers. In particular, the use of the TCR enabled evaluation of testicular cancer, an early-onset malignancy that remains relatively uncommon in Asian populations and could not be adequately assessed in the previous study. This provides a unique opportunity to investigate whether early-life DDT exposure may contribute to testicular cancer risk in a low-incidence population. We hypothesized that reproductive organs may be particularly susceptible to the long-term effects of DDT because of their dependence on tightly regulated hormonal signaling during critical developmental periods. By focusing on reproductive system cancers, this study aims to provide a more robust evaluation of the long-term impact of early-life endocrine disruption on cancer risk and to further clarify the lifelong health effects of endocrine-disrupting chemicals.

## 2. Materials and Methods

### 2.1. Study Population

The study population comprised a nationwide birth cohort of individuals born between 1952 and 1958. Although the intensive DDT indoor residual spraying campaign in Taiwan concluded in 1957, the 1958 birth cohort was included to account for potential in utero exposure during the final year of the campaign. Cancer cases occurring in this cohort between 1979 and 2022 were identified using the Taiwan Cancer Registry. Follow-up began in 1979, when individuals were approximately 21–27 years old, and continued through 2022, when they reached 64–70 years of age, enabling a longitudinal assessment of cancer incidence from early adulthood into later life.

### 2.2. Data Sources

#### 2.2.1. Cancer Data

Cancer data were obtained from the Taiwan Cancer Registry (TCR), a nationwide, population-based cancer surveillance system established in 1979. The TCR collects information on newly diagnosed cancer cases reported by hospitals with more than 50 beds across Taiwan [24]. Previous studies have reported the high quality and accuracy of the TCR database, with cancer registry coverage exceeding 97% and high rates of morphological verification [25,26]. The registry is maintained by the Health Promotion Administration, Ministry of Health and Welfare, and the data are currently housed in the Health and Welfare Data Science Center. Access to the database requires a formal application and approval [24].

In this study, we included individuals born between 1952 and 1958 and identified incident cases of selected cancers. Variables used in the analysis included sex, age, date of diagnosis, cancer site, and registered residence, along with clinical stage information. As staging data were implemented in phases, the availability of stage information varied by cancer type: breast and cervical cancer data have been available since 2002, prostate cancer since 2008, and corpus uteri and ovarian cancers since 2009 [25]. Regarding disease classification, the TCR used the International Classification of Diseases for Oncology, Field Trial (ICD-O-FT) from 1979 to 2001, and transitioned to the International Classification of Diseases for Oncology, 3rd edition (ICD-O-3) in 2002 [25]. In this study, coding systems were harmonized across versions to ensure consistency and comparability. Cancer staging was defined according to the American Joint Committee on Cancer (AJCC) staging system, with the 6th edition applied during 2007–2009 and the 7th edition used from 2010 onward [25].

#### 2.2.2. Population Data

Population data were obtained from the Taiwan Demographic Fact Book published by the Department of Household Registration, Ministry of the Interior. These data provide year-end township-level population counts stratified by sex and five-year age groups. Records from 1979 to 2006 were available only in paper format [27] and were systematically digitized as part of this study, followed by rigorous consistency checks to ensure data quality, while electronic records have been available since 2007 [28].

In this study, annual township populations were estimated as the average of the year-end populations of the current and preceding years and were used as the denominator for incidence estimation. Registered residence at the time of cancer diagnosis or population registration was used as a proxy for an individual’s place of birth and early-life exposure to approximate their early-life environment; however, this proxy may not fully capture actual birth residence or lifetime migration patterns [29], particularly given substantial internal migration in Taiwan since the 1970s. Additionally, geographic units were harmonized across years to account for changes in township administrative boundaries. After harmonization, a total of 352 township-level administrative units were included in the analyses.

For the ecological analyses, annual township-level population counts stratified by sex and age group were linked with township-level DDT spraying records and corresponding cancer incidence data from the TCR. Cancer cases and population denominators were aggregated by township, calendar year, sex, and age group to estimate age-standardized incidence rates and relative risks across exposure categories. Therefore, the unit of analysis in this study was aggregated population-level data rather than individual-level observations.

### 2.3. Exposure Assessment

DDT exposure was assessed at the township level based on historical records of IRS implemented during Taiwan’s malaria eradication campaign. These data were retrieved from official government archives, primarily the Taiwan Provincial Government Gazette [20]. According to these records, the spraying program covered all townships on Taiwan’s main island, whereas offshore islands, including Kinmen, Matsu, and Penghu, were excluded due to military administrative considerations. IRS was generally conducted once annually during the campaign period. The selection of townships for spraying each year was determined according to malaria endemicity as well as available financial and operational resources. Historically, areas with higher malaria endemicity, particularly mountainous townships, underwent more frequent spraying cycles. The cumulative number of spraying rounds recorded for each township as of 1979 was used as a proxy for environmental exposure intensity. Exposure was initially categorized into six levels (0, 1, 2, 3, 4, and 5 rounds) [30,31,32,33,34,35,36].

### 2.4. Covariates

In this study, we adjusted for age group, calendar year of diagnosis, and township-level urbanization as covariates. Age group accounted for variation in baseline cancer risk across the life course, while calendar year controlled for temporal trends in cancer incidence, including changes in screening practices and diagnostic improvements. Township-level urbanization, classified according to Liu et al. [37], served as a proxy for socioeconomic status, healthcare accessibility, and environmental conditions, thereby reducing potential confounding. Urbanization was categorized into four levels, ranging from Level I (most urbanized) to Level IV (least urbanized), based on population density, educational attainment, age structure, agricultural employment, and healthcare resource availability [37]. Due to the ecological nature of this study, individual-level covariates were not available and therefore were not included in the analysis.

### 2.5. Statistical Analysis

Descriptive statistics were used to summarize the baseline characteristics of the study population according to the frequency of township-level DDT IRS. Age-standardized incidence rates (ASIRs) for site-specific reproductive cancers were calculated using the direct standardization method, with the 2000 World Standard Population as the reference [38].

To evaluate the association between DDT exposure and cancer risk, Poisson regression models were used to estimate relative risks (RRs) and 95% confidence intervals (CIs). To assess the directionality of the association, the number of DDT spraying cycles was treated as a continuous variable under the hypothesis that increasing exposure intensity would be associated with progressively higher cancer risk.

Given that the study period spanned several decades, population migration may have introduced exposure misclassification [29], potentially affecting the accuracy and stability of the estimated associations. To partially evaluate the potential influence of migration-related exposure misclassification, additional restricted analyses were conducted excluding highly urbanized townships with substantial population inflow, which primarily corresponded to townships with zero spraying rounds [23,29]. These analyses were intended as exploratory sensitivity analyses rather than primary analyses. Because these restricted analyses excluded highly urbanized townships with substantial population inflow, the reference group differed from that used in the primary analyses. Therefore, these analyses should be interpreted as comparisons across exposure gradients among exposed areas rather than direct comparisons between exposed and unexposed populations. In addition, to explore potential nonlinear associations, exposure was categorized into three levels: low (1–2 sprayings), medium (3 sprayings), and high (4–5 sprayings), with the low-exposure group serving as the reference.

Stratified analyses by stage at diagnosis were conducted to explore whether higher levels of DDT exposure were associated with differences in stage distribution at diagnosis. According to the AJCC staging system, stages 0–II were classified as localized-stage disease, whereas stages III–IV were classified as advanced-stage disease. These analyses were restricted to periods when staging information was available in the national cancer registry, beginning in 2002 for breast and cervical cancers, 2008 for prostate cancer, and 2009 for corpus uteri and ovarian cancers. Cases with incomplete information on key variables required for analysis were excluded; however, missing data were minimal in the nationwide registry databases used in this study, and no imputation procedures were performed. All statistical analyses were performed using SAS version 9.4, with a two-sided significance level of *p* < 0.05.

The study protocol was reviewed and approved by the Research Ethics Committee of the National Health Research Institutes (Approval No. EC1110702-E). All data used in this study were obtained from a legally authorized national database. The dataset was fully de-identified prior to analysis, ensuring that no individual could be directly or indirectly identified.

## 3. Results

### 3.1. Baseline Characteristics and Cancer Distribution

Baseline characteristics of the study population in 1979 are presented in Table 1. Most participants resided in townships with three rounds of DDT spraying (*n* = 1,151,292), whereas approximately 12.5% resided in areas with no DDT spraying. A clear spatial pattern was observed in relation to urbanization level. Among townships without DDT spraying, 71.9% of the population resided in highly urbanized areas (Level I). In contrast, a higher proportion of populations in townships with the highest spraying frequency (5 rounds) resided in less urbanized areas (Levels III and IV).

Between 1979 and 2022, a total of 87,843 female and 21,401 male reproductive cancer cases were identified (Table 2). Among women, breast cancer was the most common malignancy (*n* = 53,652), with more than half of cases (53.3%) diagnosed at age ≥55 years. Among men, prostate cancer accounted for the majority of cases (*n* = 20,721), with most diagnoses (92.7%) occurring between 2011 and 2022. In contrast, only 256 men were diagnosed with testicular cancer during the study period.

### 3.2. DDT Exposure and Age-Standardized Incidence Rates

Age-standardized incidence rates (ASIRs) varied across levels of DDT spraying frequency (Table 3). Breast cancer consistently showed the highest ASIRs across all exposure groups. For hormone-related cancers, including breast, corpus uteri, and prostate cancers, the ASIRs exhibited some variation across exposure categories but did not demonstrate a consistent dose–response relationship. In contrast, ASIRs for cervix uteri cancer remained relatively stable across all exposure levels.

### 3.3. DDT Exposure Intensity and Cancer Risk

**Figure 1:**
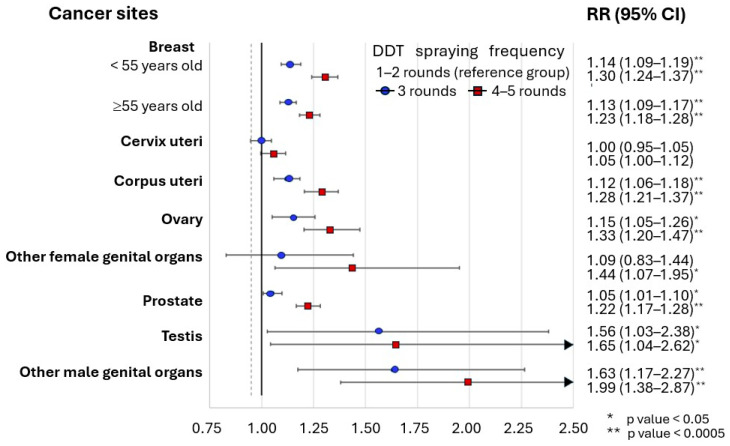
Adjusted relative risks (RRs) for site-specific cancers according to DDT exposure levels, using the low-exposure group (1–2 spraying rounds) as the reference category.

After adjustment for age group, calendar year of diagnosis, and urbanization level, Poisson regression analyses indicated positive associations between DDT exposure and several reproductive cancers (Table 4 and Figure 1).

When DDT spraying frequency was modeled as a continuous variable, each additional spraying round was significantly associated with an increased risk of multiple cancer sites (Table 4). Elevated risks were observed for several hormone-related cancers, including breast, cervical, corpus uteri, ovarian, and prostate cancers. A statistically significant association was also observed for testicular cancer (RR = 1.16, 95% CI: 1.01–1.23), although the number of cases was relatively small.

In sensitivity analyses restricted to townships with 1–5 spraying rounds, the overall pattern of positive associations remained generally consistent with the primary analyses (Table 4). Effect estimates were generally higher across most cancer sites compared with the main analysis; however, these differences may reflect changes in population composition and exposure distribution after excluding highly urbanized townships with substantial population inflow. For example, the RR for ovarian cancer increased to 1.10 (95% CI: 1.07–1.12), and similar upward shifts were observed for breast, corpus uteri, and prostate cancers. In contrast, the effect estimate for testicular cancer was slightly attenuated in the restricted analysis (RR = 1.13, 95% CI: 1.001–1.27), although the association remained positive. Overall, positive associations between DDT spraying frequency and reproductive cancer risks were observed in both the primary and restricted analyses, although the magnitude of the estimates differed between analytical approaches. Therefore, the restricted analyses should be interpreted cautiously as exploratory comparisons within exposed areas rather than evidence of reduced bias or improved exposure classification.

In categorical analyses, risk estimates increased with higher levels of DDT exposure, suggesting a dose–response relationship (Figure 1). Across most cancer sites, the highest exposure group (4–5 sprayings) consistently exhibited greater risks compared with both the low-exposure (1–2 sprayings) and the intermediate group (3 sprayings). Compared with the low-exposure group, elevated risks were observed for breast, corpus uteri, and ovarian cancers, with the strongest increase seen for ovarian cancer. Notably, the association appeared more pronounced for breast cancer diagnosed before age 55 than for later-onset breast cancer. Among male cancers, prostate cancer showed a modest increase in risk, whereas stronger associations were observed for testicular cancer (RR = 1.65, 95% CI: 1.04–2.62) and other male genital organ cancers (RR = 1.99, 95% CI: 1.38–2.87). A similar increasing pattern was observed for cervical cancer; however, the estimates did not reach statistical significance.

### 3.4. Stratified Analysis by Cancer Stage

Stratified analyses by stage at diagnosis indicated that DDT exposure was associated with cancers diagnosed at both localized and advanced stages (Table 5). For cancers diagnosed at localized stages (Stage 0–II), each additional spraying round was significantly associated with increased risks of breast, corpus uteri, ovarian, and prostate cancers. In contrast, for cancers diagnosed at advanced stages (Stage III–IV), statistically significant associations were primarily observed for corpus uteri cancer (RR = 1.07, 95% CI: 1.01–1.15), whereas associations for other cancer sites were generally weaker or not statistically significant. In analyses using the low-exposure group (1–2 spraying rounds) as the reference category, higher DDT exposure was associated with a higher proportion of cancers being diagnosed at advanced stages for breast cancer among women aged ≥55 years and for corpus uteri cancer, whereas no significant differences in stage distribution were observed for other cancer types (Table 6).

## 4. Discussion

In this ecological study, we found positive associations between DDT IRS and several reproductive cancers. Notably, increased risks were observed for both testicular and cervical cancers, for which epidemiological evidence in Asian populations remains limited. In addition, higher DDT exposure levels were associated with a higher proportion of breast and corpus uteri cancers being diagnosed at advanced stages, particularly among women aged ≥55 years. However, these findings should be interpreted cautiously given the ecological study design and the potential influence of exposure misclassification and residual confounding.

Compared with our previous study based on hazard ratios using data from the Taiwan National Health Insurance Research Database (2000–2022) [23], the present study, which followed the same population using the Taiwan Cancer Registry (1979–2022), showed a broadly consistent pattern across major reproductive cancers. Positive associations with DDT IRS exposure were observed for breast, corpus uteri, ovarian, and prostate cancers in both analyses. Notably, ovarian cancer in females and prostate cancer in males consistently exhibited the strongest associations within each sex. Overall, effect estimates were modestly higher in the present study. This increase may reflect differences in follow-up period and study design, as the extended observation window in the current analysis may have captured a greater number of early-onset cases.

Several differences from the previous study were also observed. First, associations with testicular and cervical cancers, which were not apparent in the previous study, emerged in the present analysis. This difference may be partly explained by the substantially increased number of cases, with testicular cancer cases rising from 136 to 256 and cervical cancer cases from 7540 to 14,154, representing a near doubling of case numbers and thereby improving statistical power to detect these associations. Second, while earlier findings suggested a stronger association between DDT exposure and breast cancer among women aged ≥55 years [23], the present study showed a more pronounced association for breast cancer diagnosed before age 55. This shift may be attributable to improved detection of early-onset cases under the extended follow-up period.

The observed association with testicular cancer in this study is consistent with previous epidemiologic evidence. Cohn et al. (2010) conducted a nested case–control study within the Child Health and Development Studies cohort and reported that a higher ratio of p,p′-DDT to p,p′-DDE in maternal serum was significantly associated with an increased risk of testicular cancer in male offspring decades later [39]. In utero exposure plays an important role in the etiology of testicular cancer [19,40]. Evidence suggests that the risk of testicular cancer is strongly influenced by the timing of environmental exposures, particularly maternal exposure to organochlorine pesticides (OCPs) during pregnancy [19]. In contrast, exposures occurring during adulthood appear to have weaker, less consistent associations [19]. From a mechanistic perspective, DDT has been shown to act as an androgen receptor antagonist [7], and the developing testis is particularly sensitive to EDCs during key developmental stages [41]. Early-life exposure may therefore disrupt germ cell differentiation and maturation, increasing the likelihood of malignant transformation later in life [41,42]. Notably, our findings were observed in an Asian population with a relatively low baseline incidence of testicular cancer [43], where epidemiological evidence remains limited. These findings are consistent with the hypothesis that early-life environmental exposures may contribute to testicular carcinogenesis.

Moreover, we found a positive association between early-life DDT exposure and cervical cancer risk. Epidemiologic evidence suggests that exposure to environmental pollutants may contribute to cervical carcinogenesis. Priyadarshini et al. reported that concentrations of organochlorine pesticides (OCPs), including DDT and its metabolites, were significantly higher in both serum and cervical tissue samples of cervical cancer patients compared with controls [44]. In addition, other studies have observed elevated levels of polychlorinated biphenyls (PCBs) in cervical cancer patients [45]. Higher concentrations of phthalate metabolites, particularly mono-(2-ethylhexyl) phthalate (MEHP), have also been detected in urine samples of women with cervical cancer who were infected with human papillomavirus (HPV) [46]. Experimental evidence further supports a potential biological mechanism. In vitro studies have demonstrated that o,p’-DDT, even at low doses, can directly influence cancer cell behavior by disrupting cell cycle regulation and intracellular signaling pathways [47]. Although persistent infection with HPV is the primary etiological factor for cervical cancer, environmental exposures with estrogenic activity have been proposed as important cofactors that may promote tumor initiation and progression [46]. To our knowledge, epidemiological evidence linking early-life exposure to cervical cancer remains limited. Although the effect size observed in the present study was small (RR = 1.01), the association suggests that exposure during critical developmental windows may play a role in cervical carcinogenesis. Further research is warranted to clarify the underlying mechanisms and to confirm these findings.

In this study, we observed a dose–response relationship between DDT exposure and cancers diagnosed at localized stages (Stage 0–II). In contrast, associations for cancers diagnosed at advanced stages (Stage III–IV) were less consistent and were primarily observed for corpus uteri cancer and breast cancer among women aged ≥55 years. The reasons for these differences remain unclear, and epidemiological evidence regarding environmental exposures and stage at diagnosis is still limited. Previous experimental and epidemiological studies have suggested that endocrine-disrupting chemicals (EDCs) may influence tumor behavior and disease progression through multiple biological pathways, including effects on cellular proliferation, immune regulation, and the tumor microenvironment [48,49]. At high doses, EDCs may also exert estrogen-like effects that contribute to uncontrolled cellular proliferation [50,51,52]. However, stage at diagnosis is influenced by many factors beyond environmental exposures, including genetic susceptibility [40,53], healthcare accessibility [54,55] and screening practices [45,46], diagnostic intensity, and lifestyle factors [43,56]. Therefore, the present findings should be interpreted cautiously and as exploratory analyses of stage at diagnosis rather than evidence of stage-specific cancer development or tumor progression. Further studies are needed to clarify the underlying mechanisms and potential causal relationships.

This study faced the common challenge of spatiotemporal misclassification inherent in ecological designs when assessing early-life exposure [29]. Because complete residential histories from the 1950s onward were unavailable, residential location at diagnosis was used as a proxy for historical exposure. Although this approach is commonly used in environmental epidemiology, substantial internal migration in Taiwan since the 1970s should be considered. Such migration has been largely directional, with individuals moving from rural areas with historically higher spraying intensity to more urbanized areas with lower DDT exposure [57,58]. This pattern may result in misclassification of highly exposed individuals into lower exposure categories [58]. From an epidemiologic perspective, this type of misclassification would be expected to bias estimates toward the null, suggesting that the observed relative risks may underestimate the true association [59]. To address this limitation, we conducted additional restricted analyses excluding highly urbanized townships with substantial population inflow. This approach may partially improve exposure classification related to migration patterns [60]. Associations between higher DDT exposure and stage at diagnosis remained generally consistent in this subgroup. The consistency of findings across analyses supports the robustness of the observed associations and may reduce concerns regarding migration-related exposure misclassification [61,62]. However, these exploratory analyses do not possess the same counterfactual contrast as the primary analyses due to the absence of the unexposed baseline group, which represents a major interpretational limitation. Consequently, they do not eliminate the inherent limitations of ecological exposure assignment and cannot fully address the possibility of ecological bias; thus, residual misclassification and residual confounding cannot be ruled out, and these findings should not be overinterpreted.

## 5. Strengths and Limitations

This study has several strengths. It used a nationwide cohort with more than four decades of follow-up, allowing robust evaluation of the long-term association between early-life DDT exposure and cancer risk. To our knowledge, this study is among the few in Asia that have systematically examined the long-term effects of DDT exposure during critical developmental windows. In addition, the use of a high-coverage national cancer registry provided sufficient statistical power to assess both common and rare cancers [25]. The inclusion of cancer stage at diagnosis extends prior research focused on incidence alone and provides additional information on differences in stage at diagnosis across exposure groups. Finally, the relatively homogeneous population and the use of restriction analyses provided additional context for interpreting the consistency of findings across different analytical approaches.

Several limitations should be considered. First, this study used an ecological design, with exposure assigned at the township level rather than the individual level. Because individual-level data were unavailable, caution is needed when interpreting group-level findings at the individual level. Second, internal migration in Taiwan may have introduced exposure misclassification, as residential location at diagnosis was used as a proxy for early-life exposure. Although such misclassification may have affected the accuracy and precision of the estimated associations, the direction and magnitude of the resulting bias are difficult to determine. To further explore the potential influence of migration-related exposure misclassification, we conducted additional restricted analyses focusing on non-urban populations. Because these restricted analyses excluded highly urbanized townships with substantial population inflow (primarily townships with 0 spraying rounds), the reference group differed from that used in the primary analyses, and the observed exposure gradients should therefore be interpreted cautiously. In addition, although exposure categories were based on the cumulative number of township-level spraying cycles, the timing of spraying may have differed across townships. Therefore, individuals born in later years may have experienced lower levels of direct in utero or early-life exposure in some areas. Although DDT is highly persistent in the environment and human tissues, this temporal variability may still have introduced additional exposure misclassification, potentially affecting the estimated associations. Third, exposure assessment relied on historical spraying records and did not include biological measurements of DDT or its metabolites to confirm internal exposure levels. Fourth, important individual-level confounders, including reproductive history, hormone use, obesity, smoking, alcohol consumption, HPV infection status, occupational exposures, family history, and genetic susceptibility, were unavailable in the registry databases and may have contributed to residual confounding. In addition, township-level urbanization was used as a proxy for socioeconomic status and healthcare accessibility, which may not fully capture individual-level or regional differences in screening uptake, diagnostic intensity, health literacy, and healthcare-seeking behavior. Although adjustment for calendar year may partially account for temporal changes in cancer screening and diagnostic practices, spatial heterogeneity across regions may still remain. Finally, detailed cancer staging data were only available from 2002 onward, which may introduce selection bias in analyses stratified by stage at diagnosis and limit interpretation of earlier cases.

## 6. Implications and Future Directions

Our findings suggest potential long-term environmental health implications of early-life DDT exposure. Although large-scale DDT use has become a historical practice, its environmental persistence and bioaccumulation may continue to pose long-term health concerns. Early-life exposure during critical developmental windows may influence long-term health trajectories later in life [54]. Future research should integrate biomarkers and epigenetic data and further investigate potential intergenerational and transgenerational effects to better elucidate underlying biological mechanisms and long-term health impacts across generations.

## 7. Conclusions

This study utilized Taiwan’s unique historical context in the 1950s to examine the long-term associations between early-life DDT exposure and reproductive cancers. Early-life exposure was associated with increased risks of several hormone-related cancers, with novel findings observed for testicular and cervical cancers. With extended follow-up, these findings support the hypothesis that environmental exposures during critical developmental windows may contribute to carcinogenesis across the life course, consistent with the Developmental Origins of Health and Disease (DOHaD) framework.

Although the ecological study design, potential exposure misclassification, and unavailable individual-level confounders limit causal interpretation, the generally consistent findings across analyses support the overall stability of the observed associations. From a public health perspective, these findings highlight the importance of considering the long-term health implications of persistent environmental pollutants and early-life environmental exposures. Further studies integrating biomarkers, molecular tumor characteristics, and multi-omics approaches are needed to clarify underlying biological mechanisms and potential intergenerational effects.

## Figures and Tables

**Table 1:** Baseline characteristics of the 1952–1958 birth cohort by township-level DDT indoor residual spraying frequency (1979).

	DDT Indoor Residual Spraying Frequency					
	0	1	2	3	4	5
Total population (*N*)	313,415	395,047	13,588	1,151,292	281,530	339,170
Sex						
Man (%)	49.9	51.6	52.3	50.6	51.9	52.7
Woman (%)	50.1	48.4	47.7	49.4	48.1	47.3
Age in 1979 (%)						
20–24	58.2	60.8	61.8	59.8	61.1	63.1
25–29	41.8	39.2	38.2	40.2	38.9	36.9
Urbanization ^1^						
Level I (%)	71.9	33.9	0.0	19.4	3.3	0.0
Level II (%)	28.1	22.6	0.0	47.8	57.7	33.3
Level III (%)	0.0	28.3	45.5	23.1	23.4	35.1
Level IV (%)	0.0	15.1	54.5	9.7	15.6	31.6

^1^ Urbanization was classified according to the township-level urbanization categories for Taiwan proposed by Liu et al. [37] in 2006. DDT = dichlorodiphenyltrichloroethane; *N* = number.

**Table 2:** Distribution of reproductive cancer cases diagnosed between 1979 and 2022 among individuals born in Taiwan between 1952 and 1958, by sex.

Reproductive Cancer Cases (Person)	Man	Woman
Total *N*	21,401	87,843
Age at diagnosis (years old)		
<45 (%)	164 (0.8)	12,874 (14.7)
45–55 (%)	1006 (4.7)	28,126 (32.0)
56–71 (%)	20,231 (94.5)	46,843 (53.3)
Year at diagnosis of cancer		
1979–1990 (%)	81 (0.4)	2651 (3.0)
1991–2000 (%)	125 (0.6)	14,387 (16.4)
2001–2010 (%)	1359 (6.4)	26,557 (30.2)
2011–2022 (%)	19,836 (92.7)	44,248 (50.4)
Cancer site (ICD-O-3)		
Female reproductive organs (C50–C58)		
Breast (C50)		
<55 years old	--	21,892
≥55 years old	--	31,760
Cervix uteri (C53)	--	14,154
Corpus uteri (C54–C55)	--	12,304
Ovary (C56)	--	6993
Other female genital organs (C51–C52, C57–C58)	--	740
Male reproductive organs (C60–C63)		
Prostate (C61)	20,721	--
Testis (C62)	256	--
Other male genital organs (C60, C63)	424	--
Cancer stage at diagnosis (%) ^1^		
Stage 0	0.0	5.0
Stage I	13.0	41.3
Stage II	37.5	31.5
Stage III	23.3	11.9
Stage IV	26.2	10.3

^1^ Staging information was recorded based on the American Joint Committee on Cancer (AJCC) staging system version applicable to each year of diagnosis. *N* = number; ICD-O-3 = International Classification of Diseases for Oncology, 3rd edition. -- = not applicable.

**Table 3:** Age-standardized incidence rate (ASIRs) for reproductive cancer by DDT spraying frequency (per 100,000 population).

Cancer Site (ICD-O-3)	DDT Indoor Residual Spraying Frequency					
	0	1	2	3	4	5
Female reproductive organs (C50–C58)						
Breast (C50)	8.13	7.05	5.55	7.37	7.76	7.11
<55 years old	6.49	5.73	4.50	5.91	6.38	5.67
≥55 years old	14.13	11.84	7.36	12.74	13.34	12.17
Cervix uteri (C53)	1.89	2.13	2.57	2.03	2.21	2.12
Corpus uteri (C54–C55)	1.43	1.29	1.09	1.31	1.45	1.33
Ovary (C56)	0.97	0.80	0.65	0.85	0.87	0.83
Other female genital organs (C51–C52, C57–C58)	0.08	0.10	0.10	0.09	0.10	0.10
Male reproductive organs (C60–C63)						
Prostate (C61)	1.94	1.74	1.24	1.74	1.97	1.68
Testis (C62)	0.03	0.03	0.00	0.05	0.04	0.05
Other male genital organs (C60, C63)	0.05	0.04	0.03	0.05	0.07	0.05

DDT = dichlorodiphenyltrichloroethane; ICD-O-3 = International Classification of Diseases for Oncology, 3rd edition.

**Table 4:** Relative risks of site-specific cancers associated with each additional DDT indoor residual spraying, stratified by township spraying coverage.

Cancer Site (ICD-O-3)	All Townships (0–5 Rounds)	Restricted Analysis (1–5 Rounds)
	RR (95% CI)	RR (95% CI)
Female reproductive organs (C50–C58)		
Breast (C50)		
<55 years old	1.05 (1.04–1.07) **	1.07 (1.06–1.09) **
≥55 years old	1.04 (1.03–1.05) **	1.06 (1.05–1.08) **
Cervix uteri (C53)	1.01 (1.002–1.02) *	1.01 (1.003–1.03) *
Corpus uteri (C54–C55)	1.05 (1.03–1.06) **	1.07 (1.06–1.09) **
Ovary (C56)	1.05 (1.03–1.07) **	1.10 (1.07–1.12) **
Other female genital organs (C51–C52, C57–C58)	1.08 (1.02–1.14) *	1.09 (1.02–1.17) *
Male reproductive organs (C60–C63)		
Prostate (C61)	1.03 (1.03–1.05) **	1.06 (1.04–1.07) **
Testis (C62)	1.16 (1.01–1.23) *	1.13 (1.001–1.27) **
Other male genital organs (C60, C63)	1.12 (1.04–1.21) *	1.18 (1.07–1.30) *

* *p* value < 0.05; ** *p* value < 0.0005. DDT = dichlorodiphenyltrichloroethane; ICD-O-3 = International Classification of Diseases for Oncology, 3rd edition; RR = relative risk; CI = confidence interval.

**Table 5:** Relative risks of selected site-specific cancers associated with each additional DDT indoor residual spraying cycle, stratified by stage at diagnosis.

Cancer Site (ICD-O-3)	Stage at Diagnosis	
	Localized (Stage 0–II)	Advanced (Stage III–IV)
	RRs (95% CI)	RRs (95% CI)
Breast (C50)		
<55 years old	1.07 (1.03–1.11) **	1.04 (0.96–1.13)
≥55 years old	1.08 (1.07–1.10) **	1.03 (1.00–1.07)
Cervix uteri (C53)	1.02 (0.98–1.06)	1.03 (0.98–1.09)
Corpus uteri (C54–C55)	1.05 (1.02–1.09) **	1.07 (1.01–1.15) *
Ovary (C56)	1.11 (1.03–1.20) *	1.05 (1.00–1.11)
Prostate (C61)	1.08 (1.05–1.11) **	1.01 (0.98–1.03)

* *p* value < 0.05; ** *p* value < 0.0005. RRs were estimated per additional DDT spraying cycle. DDT = dichlorodiphenyltrichloroethane; ICD-O-3 = International Classification of Diseases for Oncology, 3rd edition; RR = relative risk; CI = confidence interval. Localized stage was defined as AJCC stage 0–II, and advanced stage was defined as AJCC stage III–IV. RRs were estimated per additional DDT spraying cycle.

**Table 6:** Relative risks of selected site-specific cancers associated with moderate and high DDT indoor residual spraying, stratified by cancer stage.

Cancer Site (ICD-O-3)	Stage	Moderate Exposure (3 Rounds)	High Exposure (4–5 Rounds)
		RR (95% CI)	RR (95% CI)
Breast (C50)			
<55 years old	Localized (0–II)	1.05 (0.94–1.19)	1.31 (1.14–1.49) **
	Advanced (III–IV)	0.95 (0.75–1.22)	1.15 (0.86–1.53)
≥55 years old	Localized (0–II)	1.10 (1.05–1.15) **	1.26 (1.20–1.33) **
	Advanced (III–IV)	1.19 (1.07–1.32) **	1.13 (1.01–1.28) *
Cervix uteri (C53)	Localized (0–II)	0.98 (0.86–1.11)	1.03 (0.90–1.19)
	Advanced (III–IV)	1.23 (1.02–1.48) *	1.09 (0.88–1.34)
Corpus uteri (C54–C55)	Localized (0–II)	1.09 (0.98–1.20)	1.18 (1.05–1.32) **
	Advanced (III–IV)	1.22 (0.99–1.50)	1.33 (1.05–1.68) *
Ovary (C56)	Localized (0–II)	1.33 (1.04–1.70) *	1.45 (1.10–1.92) *
	Advanced (III-IV)	1.01 (0.85–1.19)	1.13 (0.93–1.38)
Prostate (C61)	Localized (0-II)	1.03 (0.95–1.11)	1.26 (1.15–1.37) **
	Advanced (III-IV)	1.01 (0.93–1.09)	1.06 (0.97–1.15)

* *p* value < 0.05; ** *p* value < 0.0005. The low-exposure group (1–2 spraying rounds) was used as the reference category. DDT = dichlorodiphenyltrichloroethane; ICD-O-3 = International Classification of Diseases for Oncology, 3rd edition; RR = relative risk; CI = confidence interval. Localized stage was defined as AJCC stage 0–II, and advanced stage was defined as AJCC stage III–IV. Reference group: low exposure (1–2 sprayings).

## Data Availability

The current analysis was based on data provided by the Health and Welfare Data Science Center, Ministry of Health and Welfare, Executive Yuan, Taiwan, where access is restricted to approved applicants; therefore, the data cannot be shared.

## Abbreviations

DDT	Dichlorodiphenyltrichloroethane
IRS	Indoor Residual Spraying
EDC	Endocrine-Disrupting Chemical
TCR	Taiwan Cancer Registry
AJCC	American Joint Committee on Cancer
